# Supplementary material for: Factors affecting contraceptive choice in women over 40: a qualitative study
Source: BMJ Open. 2022 Nov 22;12(11):e064987. doi: 10.1136/bmjopen-2022-064987 (PMC9685181; doi:10.1136/bmjopen-2022-064987)
Supplement: Supplementary data [file bmjopen-2022-064987supp001.pdf]

Appendix 1

Discussion Guide: Factors affecting contraceptive choice in women over 40: A Qualitative Study

Confidentiality:
Are you somewhere we can speak in private?
Everything will be kept confidential unless you or someone else is in danger of harm.
We will use made up names in the report so you cannot be identified.
Please have your mobile on silent. Let me know if you would like a break or have to take a call. You may turn off video if you would feel more comfortable.

Explain consent form and gain consent through the chat function.

1. Participant background
Can you tell be a bit about yourself?
Why did you decide to help with this research project?
Age
Studies/work/training/qualifications
What area do you live in?
Do you live with anyone else?

2. Current contraceptive use
Do you need contraception at the moment?
Are you currently in a relationship?
What type of contraception do you usually use?
How have you decided what method of contraception to use?
What are your priorities when choosing a contraceptive method?
What are your main concerns when choosing a contraceptive method?
Do you feel your priorities and /or concerns have changed over time?

3. Information
Where do you get information about contraception?
Have you previously used online sources for contraception advice?
Do you feel you've been given enough information about your contraceptive method in the past?
Have you been given updated information about contraception?

4. Preventing pregnancy
Do you worry about unplanned pregnancy?
Is preventing pregnancy your primary concern when choosing a contraceptive method?
How would an unplanned pregnancy affect you at this point in your life?

5. Symptom control

Do you/have you experience/d any menopausal symptoms e.g. change in bleeding pattern, hot sweats, change in mood?

- If yes, how do they affect you?

Have you sought advice on how to manage these symptoms?

- If yes, where did you go and did you feel informed and supported?

Have you ever used a method of contraception to help manage these symptoms?

6. Your health

Have you ever had to change contraception because of your age or health?

Do you have any medical conditions that make choosing contraception more complicated?
