# Supplementary material for: Factors affecting contraceptive choice in women over 40: a qualitative study
Source: BMJ Open. 2022 Nov 22;12(11):e064987. doi: 10.1136/bmjopen-2022-064987 (PMC9685181; doi:10.1136/bmjopen-2022-064987)
Supplement: Author's manuscript [file bmjopen-2022-064987.draft_revisions.pdf]

BMJ Open

BMJ Open is committed to open peer review. As part of this commitment we make the peer review history of every article we publish publicly available.

When an article is published we post the peer reviewers' comments and the authors' responses online. We also post the versions of the paper that were used during peer review. These are the versions that the peer review comments apply to.

The versions of the paper that follow are the versions that were submitted during the peer review process. They are not the versions of record or the final published versions. They should not be cited or distributed as the published version of this manuscript.

BMJ Open is an open access journal and the full, final, typeset and author-corrected version of record of the manuscript is available on our site with no access controls, subscription charges or pay-per-view fees (http://bmjopen.bmj.com).

If you have any questions on BMJ Open's open peer review process please email info.bmjopen@bmj.com

BMJ Open

Factors affecting contraceptive choice in women over 40: A qualitative study

Journal:	*BMJ Open*
Manuscript ID	bmjopen-2022-064987
Article Type:	Original research
Date Submitted by the Author:	26-May-2022
Complete List of Authors:	Burgin, Jo; University of Bristol<br>Bailey, Julia; University College London
Keywords:	QUALITATIVE RESEARCH, REPRODUCTIVE MEDICINE, SEXUAL MEDICINE

SCHOLARONE™
Manuscripts

BMJ

I, the Submitting Author has the right to grant and does grant on behalf of all authors of the Work (as defined in the below author licence), an exclusive licence and/or a non-exclusive licence for contributions from authors who are: i) UK Crown employees; ii) where BMJ has agreed a CC-BY licence shall apply, and/or iii) in accordance with the terms applicable for US Federal Government officers or employees acting as part of their official duties; on a worldwide, perpetual, irrevocable, royalty-free basis to BMJ Publishing Group Ltd ("BMJ") its licensees and where the relevant Journal is co-owned by BMJ to the co-owners of the Journal, to publish the Work in this journal and any other BMJ products and to exploit all rights, as set out in our licence.

The Submitting Author accepts and understands that any supply made under these terms is made by BMJ to the Submitting Author unless you are acting as an employee on behalf of your employer or a postgraduate student of an affiliated institution which is paying any applicable article publishing charge ("APC") for Open Access articles. Where the Submitting Author wishes to make the Work available on an Open Access basis (and intends to pay the relevant APC), the terms of reuse of such Open Access shall be governed by a Creative Commons licence – details of these licences and which Creative Commons licence will apply to this Work are set out in our licence referred to above.

Other than as permitted in any relevant BMJ Author's Self Archiving Policies, I confirm this Work has not been accepted for publication elsewhere, is not being considered for publication elsewhere and does not duplicate material already published. I confirm all authors consent to publication of this Work and authorise the granting of this licence.

For peer review only

Factors affecting contraceptive choice in women over 40: A Qualitative Study

Dr. Jo Burgin (corresponding author): Academic Clinical Fellow in General Practice, Centre for Academic Primary Care, Centre for Academic Primary Care, Bristol Medical School, University of Bristol, 39 Whatley Road, Bristol, BS8 2PS, Jo.burgin@nhs.net

Dr. Julia V Bailey, Associate Professor, Research Department of Primary Care and Population Health, University College London, Rowland Hill Street, London, NW3 2PF

For peer review only

For peer review only - http://bmjopen.bmj.com/site/about/guidelines.xhtml

ABSTRACT

Objective
To explore the views of women over 40 in choosing and using contraception, to inform how contraceptive counselling for this age group could be improved.

Design, Setting and Participants
Fourteen women aged 40-52 were recruited through social media platforms to take part in online semi-structured, in-depth interviews. Transcripts were analysed using a qualitative thematic approach.

Results
1) Fear of pregnancy was widely reported with participants still highly motivated to avoid pregnancy.
2) Changes of contraceptive method over the lifecourse were occasionally precipitated by emergent health conditions, but more often healthcare providers recommended a change in method due to age alone.
3) Many participants reported perimenopausal symptoms but were largely unaware of how hormonal contraception could be used to treat these symptoms.
4) Prior negative experiences with contraceptive methods, coercive or controlling experiences with healthcare providers, and traumatic life events, all contributed to a narrowing of contraceptive preference in later life.

Conclusion
Women over 40 may be highly motivated to avoid pregnancy. This age group may have more complex contraceptive histories with emerging perimenopausal symptoms. They are likely to have accumulated adverse experiences which can impact on their contraceptive choices. These factors need to be holistically explored by the clinician in order to aid shared decision making.

STRENGTHS AND LIMITATIONS

- Online recruitment excluded women without access to the internet
- Recruitment through social media platforms narrowed the range of potential participants, and may have oversampled women with particularly difficult contraceptive needs or experiences, and those with higher anxiety about risk of pregnancy
- The interviews and analysis were conducted by a researcher with a clinical background, which may have been beneficial in interpreting their contraceptive history, but may have influenced the type of information women were comfortable sharing.

For peer review only - http://bmjopen.bmj.com/site/about/guidelines.xhtml

INTRODUCTION

Motivators and barriers to contraception use change over the course of a woman's lifetime. Changes in motivation to avoid future pregnancies, medical history, and perimenopausal symptoms mean that women over 40 may need to review their contraception method of choice. Contraceptive counselling that takes a "one size fits all" approach may neglect the complex needs of older women.(1)

Preventing pregnancy
Women aged 45-49 are sexually active and 72% report using a contraceptive method.(2) However, women aged 40 and over have one of the highest rates of abortion compared to live births. In 2019, 31% of pregnancies in this age group ended in therapeutic abortion, which is higher than women under 20.(3) This suggests there is continuing unmet need, or that women over 40 may misjudge their pregnancy risk.(4)

Increased health risks and symptom control
Background risk of cardiovascular disease (CVD), breast cancer and other gynaecological cancers increase with age.(5) This may affect women's choice and clinical eligibility for certain contraceptives, particularly the combined hormonal methods. As women enter the perimenopause they can experience changes to their menstrual cycles or perimenopausal symptoms which may influence their method choice.(6)

Previous contraceptive experience
On average, women will try approximately three different contraceptive methods over the course of their reproductive lifetime.(7) Women who have had negative experiences with contraception are more likely to report unintended pregnancy in later reproductive life.(8)

Research in women over 40 has been heavily focused on which methods are clinically safe and effective. Qualitative research has focused on young women's experiences of contraception but neglected the specific priorities and concerns of older women.(9) This paper aims to explore the views of women over 40 in choosing and using contraception, to inform how contraceptive counselling for this age group could be improved.

METHODS

Participants, setting, recruitment
Women aged 40-55 were recruited from adverts placed on social media platforms and the research participant website "Call for participants". Twenty-three participants made contact with the primary researcher and 14 proceeded to interview following receipt of the study information leaflet and the consent form.

Data collection and analysis
Online, semi-structured, in-depth interviews were conducted between August and December 2021. Interviews lasted 45-60mins and were carried out by J Burgin, who is also a primary care doctor. Interviews were recorded on Microsoft Teams, transcribed using computer software, and subsequently checked against original recordings for accuracy. Transcripts were uploaded to nVivo12 software for coding and thematic analysis. Participant recruitment continued until no new themes were identified and data saturation was reached.

Patient and Public Involvement
Participants' views shaped new content on contraception for women over 40 on the Contraception Choices website. All participants will receive a copy of any published work.

RESULTS

Participant characteristics
Fourteen cis-gendered women aged 40-52 (average age 45) were recruited from across England, with the majority located in Southern England. Table 1 shows selected sexual and reproductive health characteristics of the participants.

For peer review only - http://bmjopen.bmj.com/site/about/guidelines.xhtml

**Age**	
Range	40-52
Mean	45
**Relationship status**	
Single	2
Different sex	11
Same sex	1
**Children (biological)**	
Yes	10
No	4
**Experiencing perimenopausal symptoms**	8
**Current use of HRT**	6
**Current contraceptive method**	
Intrauterine System (IUS)	4
Progestogen Only Pill (POP)	3
Implant	2
Copper Intrauterine Device (IUD)	1
Condoms	1
Vasectomy	1
None	2
**Previous number of contraceptive methods used**	
≤3	8
≥4	6

Table 1. Sexual and reproductive characteristics of participants

For peer review only

Participants discussed their perceived pregnancy risk, how their healthcare needs had changed over their lifecourse, and disclosed negative experiences which affected their approach to contraception.

Fear of pregnancy

Fear of pregnancy remained a key motivator in continued contraception use. Participants perceived an unintended pregnancy as a major negative life event.

Despite declining fertility in women over 40, all the participants felt pregnancy was a real possibility and remained cautious in their sexual relationships. Some mentioned women in their late 40s and 50s becoming pregnant unexpectedly. These narratives seemed to emphasise the real possibility of pregnancy, which they identified with.

Yeah, and I have heard of women that have naturally conceived twins in their 50s. OK, maybe they've been with a, probably, 20-30 year old at the time with my one example, but it could happen, couldn't it?

Participant 2, Age 50

The majority of participants had completed their families and mentioned the stress an additional child would place on not just themselves, but the rest of the family. Some participants had both children and parents as dependents, or were at a demanding juncture in their career, and so did not want to extend their responsibilities with another

For peer review only - http://bmjopen.bmj.com/site/about/guidelines.xhtml

child. Finances had also played into decisions to limit family size. Other participants did not want to become parents and knew they would have to terminate a pregnancy if their contraceptive method failed.

No, I think contraception is important. Because for example, if I had another child now, at this stage in life, number one, I certainly couldn't cope. I mean, I know I need to sleep. You know I have an elderly parent that I've helped with, and when you're up all night, that's not something I can do in my late 40s

Participant 11, Age 48

Some participants mentioned the negative physical effects of pregnancy and traumatic birth experiences as a motivator in using contraception to prevent pregnancy, and also as increasing their level of anxiety over the possibility of contraceptive failure.

…and the way that my labour went, and my induction went and everything, I don't think I could put myself through that again. I mean there were just so many things that happened and then went wrong

Participant 10, Age 43

Anxiety around unplanned pregnancy created anxiety for women and their partners. Some women described their partners checking they had taken their pills, or distrust of methods they were not familiar with, like the Intrauterine System (IUS). For participants not in longstanding relationships, this anxiety impacted their enjoyment of new relationships.

this should be fun, and actually it's just made it more scary. Petrified I was going to get pregnant again which would be horrendous…That would be a moral dilemma, from which I probably wouldn't recover because I couldn't do another child.

Participant 8, Age 52 (on starting a new relationship in her 50s)

Almost every participant in a long-term relationship had discussed the possibility of vasectomy with their partner, but the majority said that their partners were firmly opposed. This led to a feeling that the responsibility for contraception was one-sided, and that this was an additional burden that they had shouldered over the lifetime of their relationships.

Didn't even draw breath. Just went, no! Fair enough. I can't make him. You know that would have been my preferred option…I felt like I've just done three rounds of IVF and two children and I don't want any more chemicals in my system. And he was like, No.

Participant 14 Age 41

Only two participants said that their partner took responsibility for contraception and only one participant was currently relying on vasectomy as a form of contraception.

Change of contraceptive methods over the lifecourse

All but one participant had used the Combined Oral Contraceptive Pill (COCP) in their earlier contraceptive years. Increasing age, health conditions, and side effects had influenced method changes, with the majority of participants reporting current use of progestogen only methods.

Age

Many participants had started the COCP as teenagers as their first form of contraception. While some stopped taking it due to side effects, others continued to take it for decades without issue. However, many participants felt that being on hormonal contraception for long periods of time was harmful and that they either tried to take regular breaks or, as they got older, they became more concerned about associated health risks. For some this was

For peer review only - http://bmjopen.bmj.com/site/about/guidelines.xhtml

For peer review only

highlighted by doctors who advised method changes in their late 30s and early 40s. For others, hearing about the negative experiences of friends of colleagues influenced their decision to switch methods.

Well, you know with the contraceptive pill, you always know that there is some risks, like you could have a stroke, or you could have a thrombosis. Or, you know, there are risks of cancer. But when you were starting out, should we say in your 20, these risks seems small. But when you become older and you've seen people that might have gone through this, so you know they might now be disabled because they've had the thrombosis…and then the doctor says you are of an age where you shouldn't continue with contraceptive pills, you listen.

Participant 11, Age 48

Many women accepted this change in contraception as part of a natural progression in their contraceptive use, and a switch to a progestogen method or a coil was relatively seamless. However, for some women advice to change method seemed arbitrary and very little explanation had been provided prior to stopping the COCP.

I think my doctor had said previously. He's like at some point, you know we'll talk about maybe switching to progesterone only pill because you, um, "you're at that age now", or some kind of like throwaway statement that makes you go "What? What does that mean?"

Participant 4, Age 43

For those who had been satisfied with the COCP, stopping it resulted in the unmasking of perimenopausal symptoms.

I thought oh it can't be good for me to have all these hormones in me, maybe I should try and reduce that a little bit. Turns out my body really liked the hormones!

Participant 4, Age 43

Switching methods

While all participants were offered progestogen only methods or Intrauterine devices in place of the COCP, some experienced significant progestogen related side effects such as irregular bleeding, mood changes, breast tenderness and bloating. Participants also reported issues with accessing coil insertion if this was not offered in their GP surgery. This meant navigating coil providers at community hospitals and sexual health clinics, with some participants reporting they had been turned away due to their age.

Lack of support from healthcare providers

Some participants were confused at the variety of methods that had become available since they started using contraception and did not feel supported in making new decisions about contraception methods. This was compounded by a feeling that they had individual needs that were not being taken into consideration. In fact, at a time where their medical history and personal preferences were most complex, it was felt they were being treated as a homogenous group based solely on age.

Whereas in kind of (my) 20s and 30s I did feel very generic… I'm looking for contraception to have a different effect than what I would have done before.

Participant 3, Age 42

Health Conditions

For peer review only - http://bmjopen.bmj.com/site/about/guidelines.xhtml

Two participants mentioned health conditions (migraines and high blood pressure), that precipitated a change from the COCP. Both reported problems with alternative methods of contraception and relied for some period of time on withdrawal methods, which, for one participant, resulted in an unintended pregnancy.

However, other participants had health conditions which had led to a change of contraception specifically for its health benefits. Endometriosis and menorrhagia were both drivers for considering the IUS. Another participant started a progestogen method to tackle Pre-menstrual syndrome.

Perimenopause

Confusion about the interplay between the menopause and hormonal contraceptive methods was common. Participants didn't understand how they would identify the menopausal transition while taking contraceptive methods that already affected their periods. This caused frustration as it could mean they were taking contraception even when it may no longer be needed to prevent pregnancy.

And I would love not to be on the pill. I'm assuming I'm gonna hit menopause soon. More than happy to. I'm embracing it. And it really is like, bring it on. Let me get on with the rest of my life. But how do you know, if you're taking the pill? How on earth do you know?

Participant 2, Age 50

Many participants spoke about perimenopausal symptoms and the difficulties they had experienced diagnosing and treating their symptoms. While some participants had been offered the IUS to control heavy periods, or as an alternative to the COCP, few were aware that the IUS could be used as a component of Hormone Replacement Therapy (HRT). Only one participant knew that combined hormonal contraception could alleviate perimenopausal symptoms. Overall there was a sense of frustration that their hormonal health had not been treated more holistically and that perimenopausal symptoms were misdiagnosed or ignored.

I don't think anyone has ever really spoken to me about how you might want to change contraception as you do approach menopause and why.

Participant 12, Age 48

Previous Experiences of Contraception, Coercion and Trauma

Contraceptive experience

Participants had tried between 2 and 6 contraceptive methods over their lifetime. Those that had used more than 3 methods were more likely to report hormonal side effects and this made finding suitable contraception in later life problematic. Mood changes were the most commonly reported concern about trying different hormonal methods.

I'd previously been on the injection…hormone…thing, for contraception, some years previously and it sent me a little bit like, you know, insane, I can't think of a better word. So I questioned them quite heavily about that side of things because I wasn't keen to go through that again

Participant 5, Age 41

Painful coil insertions (both personal experiences and narratives from friends or family), were frequently cited as reasons for discounting intrauterine contraception in later life.

I'm a bit scared to have it put in because everybody that I've spoken to who has one, or has had one, says it really hurts. So I'm just like, I hurt enough round there

For peer review only - http://bmjopen.bmj.com/site/about/guidelines.xhtml

Participant 7, Age 50

Coercion and Trauma

Experiences of contraceptive coercion in a medical setting had severe and long-lasting effects. These incidents left participants feeling a loss of control over their own bodies and sense that their wishes had been violated. This undermined trust in both medical professionals and, by association, the treatments they were offered. Traumatic experiences influenced the type of contraception that participants felt comfortable with, namely a contraceptive that was under complete user control such as condoms and pills. It also made participants hesitant about any methods that involved intimate procedures where they may experience a similar loss of autonomy and flashbacks. Complex and traumatic birthing experiences were also reasons for declining intrauterine contraception, or for requiring sedation in order to have one fitted.

Yeah, I hated that I felt really violated by almost, almost instantly. I felt like I'd been massively pressurized into it, and it was. I couldn't get it out again…I felt yeah really out of control…. And I've always been, since I had that injection, terrified of them. Anything hormonal.

Participant 13, Age 40 (discussing contraceptive injection as a university student)

Yeah, I mean even going for a smear is a big thing. I cried through most of them just because you're in that position. Somebody is going there. It's gonna hurt. I know they say it doesn't, but it, it does. I probably cry about it now, which is ridiculous, isn't it?

Participant 8, Age 52 (following failed induction of labour)

Conversely, a few participants reported their repeated exposure to medical procedures as enabling them to manage more intimate procedures, like the insertion of an intrauterine device, with ease. The stark difference between these experiences seemed to centre around how much autonomy the participant felt they had during these procedures.

I had no problems, but I've done three rounds of IVF so I had so…sounds horrendous!...so many things put inside me, that actually this wasn't an issue.

Participant 14, Age 41

Other traumatic incidents outside the medical setting, including sexual assault and near miss attacks, were also reported influencing choice of contraception. Again, this reinforced the need for control over their body and reproductive choices.

Yeah, I've had another really traumatic experience when I was 16 when somebody almost raped me and I think maybe I felt like it was really important to be totally in control

Participant 13, Age 40

DISCUSSION

Women over 40 have distinct motivators and barriers to choosing and using contraception. Previous studies have attributed the relatively low use of reliable contraception in women over 40 to women's perceptions of themselves as low risk due to declining fertility.(4,8) However, this study found that while women recognised fertility levels declined in their 40s, they thought of pregnancy as a real possibility, and something that would have a large and detrimental impact on their lives. It created anxiety both for them as individuals, and also in their relationships.

Many participants reported stopping the COCP due to age alone. This seemed to be provider led, but is not supported by clinical guidelines. In fact, combined hormonal contraception is increasingly recognised as a useful tool in women over 40 as it provides both contraception and stable levels of oestrogen, thereby treating

For peer review only - http://bmjopen.bmj.com/site/about/guidelines.xhtml

For peer review only

perimenopausal symptoms.(10) This highlights the importance of meaningful discussions about risk and benefits in continuing with a combined hormonal method so that contraceptive options are not narrowed arbitrarily(11).

Participants valued a tailored approach to contraceptive counselling and disliked being advised on the basis of age alone, which echoes the advice in clinical guidelines.(1,12–14) Despite this, women still feel they are not treated either holistically, or as individuals. A reframing of contraception within a “hormonal health” consultation could allow more space for women to share their concerns and needs.

Many participants felt frustrated and disappointed that their healthcare provider had not helped them to identify perimenopausal symptoms earlier. Perimenopausal symptoms are not routinely discussed in contraception reviews meaning that these are missed, especially if women’s periods are already altered by hormonal contraception.(15) Participants felt they would benefit from earlier information on the perimenopause and HRT in order to inform their contraceptive decisions.

While it might be assumed that women over 40 are more experienced in using contraception, sometimes it is these experiences which create further obstacles to satisfactory contraception use. The side effects from previous contraception may make women more apprehensive to try new methods.(16–18) This is pertinent for women over 40 as they have had more exposure to adverse experiences over their reproductive lifetime. These adverse experiences both within and outside of the healthcare system extended well beyond simple contraceptive side effects.

Interactions with healthcare providers in all areas of sexual and reproductive health coloured the contraception women felt comfortable with. Experiencing coercion or control in a healthcare setting led to particularly strong negative feelings and narrowed contraceptive preferences. Adverse experiences undermined trust in methods that require provider insertion, which are typically the most long acting and reliable. Traumatic experiences outside a healthcare setting, such as sexual assault, also influenced women’s method choice. These barriers are not traditionally addressed during contraceptive counselling.(19) While they are clearly important issues to address in every age group, it could be seen as particularly pertinent for women over 40 who are more likely to have been through traumatic births and invasive gynaecological investigations. Enquiring about these types of experiences when initiating or switching contraception would be a positive step in making the consultation more tailored and holistic.

Limitations
Online recruitment excluded women without access to the internet. Recruitment through social media platforms narrowed the range of potential participants, and may have oversampled women with particularly difficult contraceptive needs or experiences, and those with higher anxiety about risk of pregnancy. The interviews and analysis were conducted by a researcher with a clinical background, which may have been beneficial in interpreting their contraceptive history, but may have influenced the type of information women were comfortable sharing.

CONCLUSION

Women over 40 may be still highly motivated to avoid pregnancy. This age group is likely to have more complex contraceptive histories and are likely to have accumulated adverse experiences which can impact on their contraceptive choices. These factors need to be holistically explored by the clinician in order to aid shared decision making.

ETHICS APPROVAL STATEMENT

Ethics approval was obtained from University College London (UCL) [Ethics no 19875/001].

COMPETING INTERESTS

None declared

For peer review only - http://bmjopen.bmj.com/site/about/guidelines.xhtml

FUNDING

Jo Burgin is a National Institute for Health Research academic fellow

CONTRIBUTORSHIP STATEMENT

JB and JoB devised the study. JoB designed the topic guide with input from JB. JoB carried out all interviews, transcription and analysis with supervision from JB. Emergent results were discussed with other researchers. JoB wrote the article with input from JB. Both authors approved the final version and are accountable for all aspects of the work.

DATA SHARING STATEMENT

Data are available upon reasonable request. Fully anonymised interview transcripts are available from Dr Jo Burgin, jo.burgin@nhs.net. Available for research purposes only.

For peer review only

For peer review only - http://bmjopen.bmj.com/site/about/guidelines.xhtml

REFERENCES

1. Roberts A, Noyes J. Contraception and women over 40 years of age: mixed-method systematic review. J Adv Nurs. 2009 Jun;65(6):1155–70.

2. FRSH. Faculty for Reproductive and Sexual Health, Clinical Guideline: Contraception for Women Aged over 40 Years (August 2017, amended September 2019). 2019.

3. ONS. Office for National Statistics, Conception Statistics, England and Wales, 2019. [Internet]. 2019. Available from: https://www .ons.gov.uk/peoplepopulationandcommunity/birthsdeathsandmarriages/conceptionandfertilityrates /datasets/conceptionstatisticsenglandandwalesreferencetables.

4. Sherman CA, Harvey SM, Noell J. "Are *They* Still Having Sex?" STIs and Unintended Pregnancy Among Mid-Life Women. J Women Aging. 2005 Sep 20;17(3):41–55.

5. Carslaw H, Cosh A. Contraception for older women. InnovAiT Educ Inspir Gen Pract. 2016 Aug;9(8):454–60.

6. Guerin J, Engelmann A, Mattamana M, Borgelt LM. Use of hormonal contraceptives in perimenopause: A systematic review. Pharmacother J Hum Pharmacol Drug Ther. 2022 Feb;42(2):154–64.

7. Daniels K, Mosher WD. Contraceptive methods women have ever used: United States, 1982-2010. Natl Health Stat Rep. 2013 Feb 14;(62):1–15.

8. Godfrey EM, Zapata LB, Cox CM, Curtis KM, Marchbanks PA. Unintended pregnancy risk and contraceptive use among women 45-50 years old: Massachusetts, 2006, 2008, and 2010. Am J Obstet Gynecol. 2016 Jun;214(6):712.e1-712.e8.

9. Alspaugh A, Barroso J, Reibel M, Phillips S. Women's Contraceptive Perceptions, Beliefs, and Attitudes: An Integrative Review of Qualitative Research. J Midwifery Womens Health. 2020 Jan;65(1):64–84.

10. Mendoza N, Sanchez-Borrego R. Classical and newly recognised non-contraceptive benefits of combined hormonal contraceptive use in women over 40. Maturitas. 2014 May;78(1):45–50.

11. Mendoza N, Soto E, Sánchez-Borrego R. Do women aged over 40 need different counseling on combined hormonal contraception? Maturitas. 2016 May;87:79–83.

12. Kim C-S, Tikhonov D, Merjanian L, Balica AC. Contraception pathway: application for midlife women. Womens Midlife Health. 2017 Dec;3(1):10.

13. Nelson AL, Cohen S, Galitsky A, Hathaway M, Kappus D, Kerolous M, et al. Women's perceptions and treatment patterns related to contraception: results of a survey of US women. Contraception. 2018 Mar;97(3):256–73.

14. Madden T, Secura GM, Nease RF, Politi MC, Peipert JF. The role of contraceptive attributes in women's contraceptive decision making. Am J Obstet Gynecol. 2015 Jul;213(1):46.e1-46.e6.

15. Marnocha SK, Bergstrom M, Dempsey LF. The lived experience of perimenopause and menopause. Contemp Nurse. 2011 Feb;37(2):229–40.

16. Godfrey EM, Chin NP, Fielding SL, Fiscella K, Dozier A. Contraceptive methods and use by women aged 35 and over: A qualitative study of perspectives. BMC Womens Health. 2011 Dec;11(1):5.

For peer review only - http://bmjopen.bmj.com/site/about/guidelines.xhtml

17. Gomez AM, Arteaga S, Aronson N, Goodkind M, Houston L, West E. No Perfect Method: Exploring How Past Contraceptive Methods Influence Current Attitudes Toward Intrauterine Devices. Arch Sex Behav. 2020 May;49(4):1367–78.

18. Alspaugh A, Reibel MD, Im E-O, Barroso J. “Since I’m a little bit more mature”: contraception and the arc of time for women in midlife. Womens Midlife Health. 2021 Dec;7(1):3.

19. Owens L, Terrell S, Low LK, Loder C, Rhizal D, Scheiman L, et al. Universal precautions: the case for consistently trauma-informed reproductive healthcare. Am J Obstet Gynecol. 2021 Aug;S0002937821008802.

For peer review only

For peer review only - http://bmjopen.bmj.com/site/about/guidelines.xhtml

"You're at that age now" Factors affecting contraceptive choice in women over 40

Standards for Reporting Qualitative Research (SRQR)*

http://www.equator-network.org/reporting-guidelines/srqr/

Title and abstract

	Page
**Title** - Concise description of the nature and topic of the study Identifying the study as qualitative or indicating the approach (e.g., ethnography, grounded theory) or data collection methods (e.g., interview, focus group) is recommended	1
**Abstract** - Summary of key elements of the study using the abstract format of the intended publication; typically includes background, purpose, methods, results, and conclusions	2

Introduction

**Problem formulation** - Description and significance of the problem/phenomenon studied; review of relevant theory and empirical work; problem statement	3
**Purpose or research question** - Purpose of the study and specific objectives or questions	3

Methods

**Qualitative approach and research paradigm** - Qualitative approach (e.g., ethnography, grounded theory, case study, phenomenology, narrative research) and guiding theory if appropriate; identifying the research paradigm (e.g., postpositivist, constructivist/ interpretivist) is also recommended; rationale**	3
**Researcher characteristics and reflexivity** - Researchers' characteristics that may influence the research, including personal attributes, qualifications/experience, relationship with participants, assumptions, and/or presuppositions; potential or actual interaction between researchers' characteristics and the research questions, approach, methods, results, and/or transferability	3
**Context** - Setting/site and salient contextual factors; rationale**	3
**Sampling strategy** - How and why research participants, documents, or events were selected; criteria for deciding when no further sampling was necessary (e.g., sampling saturation); rationale**	3
**Ethical issues pertaining to human subjects** - Documentation of approval by an appropriate ethics review board and participant consent, or explanation for lack thereof; other confidentiality and data security issues	11
**Data collection methods** - Types of data collected; details of data collection procedures including (as appropriate) start and stop dates of data collection and analysis, iterative process, triangulation of sources/methods, and modification of procedures in response to evolving study findings; rationale**	3

For peer review only
For peer review only - http://bmjopen.bmj.com/site/about/guidelines.xhtml

**Data collection instruments and technologies** - Description of instruments (e.g., interview guides, questionnaires) and devices (e.g., audio recorders) used for data collection; if/how the instrument(s) changed over the course of the study	3
**Units of study** - Number and relevant characteristics of participants, documents, or events included in the study; level of participation (could be reported in results)	3-4
**Data processing** - Methods for processing data prior to and during analysis, including transcription, data entry, data management and security, verification of data integrity, data coding, and anonymization/de-identification of excerpts	3
**Data analysis** - Process by which inferences, themes, etc., were identified and developed, including the researchers involved in data analysis; usually references a specific paradigm or approach; rationale**	3
**Techniques to enhance trustworthiness** - Techniques to enhance trustworthiness and credibility of data analysis (e.g., member checking, audit trail, triangulation); rationale**	3

Results/findings

**Synthesis and interpretation** - Main findings (e.g., interpretations, inferences, and themes); might include development of a theory or model, or integration with prior research or theory	4-8
**Links to empirical data** - Evidence (e.g., quotes, field notes, text excerpts, photographs) to substantiate analytic findings	4-8

Discussion

**Integration with prior work, implications, transferability, and contribution(s) to the field** - Short summary of main findings; explanation of how findings and conclusions connect to, support, elaborate on, or challenge conclusions of earlier scholarship; discussion of scope of application/generalizability; identification of unique contribution(s) to scholarship in a discipline or field	8-9
**Limitations** - Trustworthiness and limitations of findings	9

Other

**Conflicts of interest** - Potential sources of influence or perceived influence on study conduct and conclusions; how these were managed	9
**Funding** - Sources of funding and other support; role of funders in data collection, interpretation, and reporting	9

*The authors created the SRQR by searching the literature to identify guidelines, reporting standards, and critical appraisal criteria for qualitative research; reviewing the reference lists of retrieved sources; and contacting experts to gain feedback. The SRQR aims to improve the transparency of all aspects of qualitative research by providing clear standards for reporting qualitative research.

For peer review only

For peer review only - http://bmjopen.bmj.com/site/about/guidelines.xhtml

**The rationale should briefly discuss the justification for choosing that theory, approach, method, or technique rather than other options available, the assumptions and limitations implicit in those choices, and how those choices influence study conclusions and transferability. As appropriate, the rationale for several items might be discussed together.

Reference:

O'Brien BC, Harris IB, Beckman TJ, Reed DA, Cook DA. **Standards for reporting qualitative research: a synthesis of recommendations.** *Academic Medicine*, Vol. 89, No. 9 / Sept 2014 DOI: 10.1097/ACM.0000000000000388

For peer review only

For peer review only - http://bmjopen.bmj.com/site/about/guidelines.xhtml

BMJ Open

Factors affecting contraceptive choice in women over 40: A qualitative study

Journal:	*BMJ Open*
Manuscript ID	bmjopen-2022-064987.R1
Article Type:	Original research
Date Submitted by the Author:	06-Sep-2022
Complete List of Authors:	Burgin, Jo; University of Bristol<br>Bailey, Julia; University College London
**Primary Subject Heading**:	Sexual health
Secondary Subject Heading:	Reproductive medicine
Keywords:	QUALITATIVE RESEARCH, REPRODUCTIVE MEDICINE, SEXUAL MEDICINE

SCHOLARONE™
Manuscripts

I, the Submitting Author has the right to grant and does grant on behalf of all authors of the Work (as defined in the below author licence), an exclusive licence and/or a non-exclusive licence for contributions from authors who are: i) UK Crown employees; ii) where BMJ has agreed a CC-BY licence shall apply, and/or iii) in accordance with the terms applicable for US Federal Government officers or employees acting as part of their official duties; on a worldwide, perpetual, irrevocable, royalty-free basis to BMJ Publishing Group Ltd ("BMJ") its licensees and where the relevant Journal is co-owned by BMJ to the co-owners of the Journal, to publish the Work in this journal and any other BMJ products and to exploit all rights, as set out in our licence.

The Submitting Author accepts and understands that any supply made under these terms is made by BMJ to the Submitting Author unless you are acting as an employee on behalf of your employer or a postgraduate student of an affiliated institution which is paying any applicable article publishing charge ("APC") for Open Access articles. Where the Submitting Author wishes to make the Work available on an Open Access basis (and intends to pay the relevant APC), the terms of reuse of such Open Access shall be governed by a Creative Commons licence – details of these licences and which Creative Commons licence will apply to this Work are set out in our licence referred to above.

Other than as permitted in any relevant BMJ Author's Self Archiving Policies, I confirm this Work has not been accepted for publication elsewhere, is not being considered for publication elsewhere and does not duplicate material already published. I confirm all authors consent to publication of this Work and authorise the granting of this licence.

For peer review only

Factors affecting contraceptive choice in women over 40: A Qualitative Study

Dr. Jo Burgin (corresponding author): Academic Clinical Fellow in General Practice, Centre for Academic Primary Care, Centre for Academic Primary Care, Bristol Medical School, University of Bristol, 39 Whatley Road, Bristol, BS8 2PS, Jo.burgin@nhs.net

Dr. Julia V Bailey, Associate Professor, Research Department of Primary Care and Population Health, University College London, Rowland Hill Street, London, NW3 2PF

For peer review only

For peer review only - http://bmjopen.bmj.com/site/about/guidelines.xhtml

ABSTRACT

Objective
To explore the views of women over 40 in choosing and using contraception, to inform how contraceptive counselling for this age group could be improved.

Design, Setting and Participants
Fourteen women aged 40-52 were recruited through social media platforms to take part in online semi-structured, in-depth interviews. Transcripts were analysed using a qualitative thematic approach.

Results
1) Participants were anxious about unplanned pregnancy, and still highly motivated to avoid this.
2) Changes of contraceptive method over the lifecourse were occasionally precipitated by emergent health conditions, but healthcare providers often recommended a change in method on the basis of age alone.
3) Participants were experiencing perimenopausal symptoms but were largely unaware of how hormonal contraception could be used to treat these symptoms.
4) Prior negative experiences with contraceptive methods, coercive experiences with healthcare providers, and traumatic life events, all contributed to a narrowing of contraceptive preference in later life.

Conclusion
Women over 40 may be highly motivated to avoid pregnancy. This age group may have complex contraceptive histories with emerging perimenopausal symptoms. Women over 40 may have accumulated adverse experiences which impact their contraceptive choices. These factors need to be explored by clinicians, to facilitate shared decision making.

STRENGTHS AND LIMITATIONS

- Online recruitment excluded women without access to the internet
- Recruitment through social media platforms narrowed the range of potential participants, and may have oversampled women with particularly difficult contraceptive needs or experiences, and those with higher anxiety about unplanned pregnancy
- Recruitment through specific social media platforms and interest groups may have led to a relatively homogenous participant group that limited the variety of themes discussed
- The interviews and analysis were conducted by a researcher with a clinical background, which may have been beneficial in interpreting their contraceptive history, but may have influenced the type of information women were comfortable sharing. However, the interviewer was a woman of comparable age to the participants which may have facilitated open discussion regarding personal experiences
- Online interviews allowed recruitment from different regions of England, reflecting multiple healthcare localities and providers.

For peer review only

For peer review only - http://bmjopen.bmj.com/site/about/guidelines.xhtml

INTRODUCTION

Motivators and barriers to contraception use change over the course of a woman's life. Changes in motivation to avoid future pregnancies, medical history, and perimenopausal symptoms mean that women over 40 may need to review their contraception method of choice. Historically, contraceptive counselling has been poorly defined, and has concentrated on the clinician imparting information to provide the patient with a safe, acceptable and reliable method. However, more recently there have been attempts to define contraceptive counselling as a dynamic "exchange of information" between the patient and the provider which puts patients' needs and preferences at the heart of the consultation.(1) The majority of women over 40 are provided with contraception in primary care.(2) Lack of flexibility in appointment time, and the increasing popularity of templates in General Practice may lead to a "one size fits all" approach which neglects the complex needs of older women.(3–5)

Preventing pregnancy

Most women aged 45-49 are sexually active, and 72% report using a contraceptive method.(6) However, women aged 40 and over have one of the highest rates of abortion compared to live births. In 2019, 31% of pregnancies in this age group ended in therapeutic abortion, which is higher than women under 20.(7) This suggests there is continuing unmet need, or that women over 40 may misjudge the likelihood of pregnancy.(8)

Increased health risks and symptom control

Background risk of cardiovascular disease (CVD), breast cancer and other gynaecological cancers increase with age.(9) This may affect choice and clinical eligibility for certain contraceptive methods, particularly combined hormonal methods. As women enter the perimenopause they can experience changes to their menstrual cycles or perimenopausal symptoms which may influence their method choice.(10)

Previous contraceptive experience

On average, women will try approximately three different contraceptive methods over the course of their reproductive lifetime.(11) Women who have had negative experiences with contraception are more likely to report unintended pregnancy in later reproductive life.(12)

Research in women over 40 has been heavily focused on which methods are clinically safe and effective. Globally, quantitative data on contraception is either not collected or is routinely incomplete for women over 45 and may neglect to record IUD use or previous hysterectomy.(13) Qualitative research has focused on young women's experiences of contraception but neglected the specific priorities and concerns of older women.(14) This paper aims to explore the views of women over 40 in choosing and using contraception, to inform how contraceptive counselling for this age group could be improved.

METHODS

Participants, setting, recruitment

Women aged 40-55 were recruited from adverts on social media platforms (Twitter and Facebook) between June and November 2021. Twitter adverts were placed on the accounts of the research team and their academic departments, these were then disseminated by other academics and organisations with interests in contraception (e.g. Faculty for Reproductive and Sexual Health, Decolonising Contraception), endometriosis (e.g. Endobonds), and menopause (e.g. Menopause Café, Black Women in Menopause). Adverts were also placed on Facebook pages for Perimenopause and Menopause interest groups. Finally, a study page was set up on the research participant website "Call for participants", which recruited participants between June and December 2021. Four participants were forwarded information from social media adverts by colleagues or friends.

Twenty-three participants made contact with the primary researcher and 14 proceeded to interview following receipt of the study information leaflet and the consent form. Recruitment took place during the Covid-19 pandemic when access to healthcare was limited by intermittent lockdowns and reduced NHS capacity, particularly for non-urgent care.

Data collection and analysis

For peer review only - http://bmjopen.bmj.com/site/about/guidelines.xhtml

Online, semi-structured, in-depth interviews were conducted between August and December 2021. One to one interviews lasted 45-60mins and were carried out by J Burgin, who is also a primary care doctor. A discussion guide was used to cover broad areas of interest (see Appendix 1). Participants had the choice to have their cameras on or off for the duration of the interview: 12 participants chose to have cameras on, while two chose to have them turned off. The researcher had their camera on for all interviews. Interviews were recorded on Microsoft Teams, transcribed using computer software, and subsequently checked against original recordings for accuracy. Transcripts were uploaded to nVivo12 software for coding and thematic analysis. (15)

A combination of inductive and deductive thematic analysis was used.(16) All interviews were read a minimum of three times, the first reading identified broad themes, which were largely influenced by the topic guide. The second reading allowed for a more inductive approach, where data were coded line by line and broad, or significant themes were reworked. Finally, a third reading cross referenced themes with the data to ensure correlation and depth of meaning. Participant recruitment continued until no new themes were identified.

Patient and Public Involvement

Participants' views shaped new content on contraception for women over 40 on the Contraception Choices website. All participants will receive a copy of any published work.

RESULTS

Participant characteristics

Fourteen cis-gendered women aged 40-52 (average age 45) were recruited from across England, with the majority located in Southern England. All women were employed, or lived with a partner who was employed, were digitally literate and did not require the assistance of a translator to complete their interviews. Table 1 shows selected sexual and reproductive health characteristics of the participants.

**Age**	
Range	40-52yrs
Mean	45 yrs
**Relationship status**	
Single	2
Different gender	11
Same gender	1
**Children (biological)**	
Yes	10
No	4
**Experiencing perimenopausal symptoms**	8
**Current use of HRT**	6
**Current contraceptive method**	
Intrauterine System (IUS)	4
Progestogen Only Pill (POP)	3
Implant	2
Copper Intrauterine Device (IUD)	1
Condoms	1
Vasectomy	1
None	2
**Previous number of contraceptive methods used**	

For peer review only - http://bmjopen.bmj.com/site/about/guidelines.xhtml

2-3	8
4-5	5
6+	1
Location (England)	
South East & London	7
South West	6
North West	1

Table 1. Sexual and reproductive characteristics of participants

Themes

Participants discussed their perceived pregnancy risk, how their healthcare needs had changed over their lifecourse, and disclosed negative experiences which affected their approach to contraception.

Fear of pregnancy

Fear of pregnancy remained a key motivator in continued contraception use. Participants perceived an unintended pregnancy as a major negative life event.

Despite declining fertility in women over 40, all the participants felt pregnancy was a real possibility and remained cautious in their sexual relationships. Some mentioned women in their late 40s and 50s becoming pregnant unexpectedly. These narratives seemed to emphasise the real possibility of pregnancy, which they identified with.

Yeah, and I have heard of women that have naturally conceived twins in their 50s. OK, maybe they've been with a, probably, 20-30 year old at the time with my one example, but it could happen, couldn't it?

Participant 2, Age 50

The majority of participants had completed their families and mentioned the stress an additional child would place on, not just themselves, but the rest of the family. Some participants had both children and parents as dependents, or were at a demanding juncture in their career, and so did not want to extend their responsibilities with another child. Finances had also played into decisions to limit family size. Other participants did not want to become parents and knew they would have to terminate a pregnancy if their contraceptive method failed.

No, I think contraception is important. Because for example, if I had another child now, at this stage in life, number one, I certainly couldn't cope. I mean, I know I need to sleep. You know I have an elderly parent that I've helped with, and when you're up all night, that's not something I can do in my late 40s

Participant 11, Age 48

Some participants mentioned the negative physical effects of pregnancy and traumatic birth experiences as a motivator in using contraception to prevent pregnancy, and also as increasing their level of anxiety over the possibility of contraceptive failure.

…and the way that my labour went, and my induction went and everything, I don't think I could put myself through that again. I mean there were just so many things that happened and then went wrong

Participant 10, Age 43

Worry around unplanned pregnancy created anxiety for women and their partners. Some women described their partners checking they had taken their pills, or distrust of methods they were not familiar with, like the Intrauterine

For peer review only - http://bmjopen.bmj.com/site/about/guidelines.xhtml

System (IUS). For participants not in longstanding relationships, this anxiety impacted their enjoyment of new relationships.

this should be fun, and actually it's just made it more scary. Petrified I was going to get pregnant again which would be horrendous…That would be a moral dilemma, from which I probably wouldn't recover because I couldn't do another child.

Participant 8, Age 52 (on starting a new relationship in her 50s)

Almost every participant in a long-term relationship had discussed the possibility of vasectomy with their partner, but the majority said that their partners were firmly opposed. This led to a feeling that the responsibility for contraception was one-sided, and that this was an additional burden that they had shouldered over the lifetime of their relationships.

Didn't even draw breath. Just went, no! Fair enough. I can't make him. You know that would have been my preferred option…I felt like I've just done three rounds of IVF and two children and I don't want any more chemicals in my system. And he was like, No.

Participant 14 Age 41

Only two participants said that their partner took responsibility for contraception and only one participant was currently relying on vasectomy as a form of contraception.

Change of contraceptive methods over the lifecourse

All but one participant had used the Combined Oral Contraceptive Pill (COCP) in their earlier contraceptive years. Increasing age, health conditions, and side effects had influenced method changes, with the majority of participants reporting current use of progestogen only methods.

Age

Many participants had started the COCP as teenagers as their first form of contraception. While some stopped taking it due to side effects, others continued to take it for decades without issue. However, many participants felt that being on hormonal contraception for long periods of time was harmful and that they either tried to take regular breaks or, as they got older, they became more concerned about associated health risks. For some this was highlighted by doctors who advised method changes in their late 30s and early 40s. For others, hearing about the negative experiences of friends of colleagues influenced their decision to switch methods.

Well, you know with the contraceptive pill, you always know that there is some risks, like you could have a stroke, or you could have a thrombosis. Or, you know, there are risks of cancer. But when you were starting out, should we say in your 20, these risks seems small. But when you become older and you've seen people that might have gone through this, so you know they might now be disabled because they've had the thrombosis…and then the doctor says you are of an age where you shouldn't continue with contraceptive pills, you listen.

Participant 11, Age 48

Many women accepted this change in contraception as part of a natural progression in their contraceptive use, and a switch to a progestogen method or a coil was relatively seamless. However, for some women advice to change method seemed arbitrary and very little explanation had been provided prior to stopping the COCP.

I think my doctor had said previously. He's like at some point, you know we'll talk about maybe switching to progesterone only pill because you, um, "you're at that age now", or some kind of like throwaway statement that makes you go "What? What does that mean?"

Participant 4, Age 43

For peer review only - http://bmjopen.bmj.com/site/about/guidelines.xhtml

For those who had been satisfied with the COCP, stopping it resulted in the unmasking of perimenopausal symptoms.

I thought oh it can't be good for me to have all these hormones in me, maybe I should try and reduce that a little bit. Turns out my body really liked the hormones!

Participant 4, Age 43

While all participants were offered progestogen only methods or Intrauterine devices in place of the COCP, some experienced significant progestogen related side effects such as irregular bleeding, mood changes, breast tenderness and bloating. Participants also reported difficulty accessing coil insertion if this was not offered in their GP surgery. This meant navigating coil providers at community hospitals and sexual health clinics, with some participants reporting they had been turned away due to their age.

Switching methods

Participants had tried between 2 and 6 contraceptive methods over their lifetime. Those that had used more than 3 methods reported hormonal side effects as a reason for switching methods, and this made finding suitable contraception in later life problematic. Mood changes were the most commonly reported concern about trying different hormonal methods.

I'd previously been on the injection…hormone…thing, for contraception, some years previously and it sent me a little bit like, you know, insane, I can't think of a better word. So I questioned them quite heavily about that side of things because I wasn't keen to go through that again

Participant 5, Age 41

Painful coil insertions (both personal experiences and narratives from friends or family), were frequently cited as reasons for discounting intrauterine contraception in later life.

I'm a bit scared to have it put in because everybody that I've spoken to who has one, or has had one, says it really hurts. So I'm just like, I hurt enough round there

Participant 7, Age 50

Lack of tailored support from healthcare providers

Some participants were confused at the variety of methods that had become available since they started using contraception and did not feel supported in making new decisions about contraception methods. This was compounded by a feeling that they had individual needs that were not being taken into consideration. In fact, at a time where their medical history and personal preferences were most complex, it was felt they were being treated as a homogenous group based solely on age.

Whereas in kind of (my) 20s and 30s I did feel very generic… I'm looking for contraception to have a different effect than what I would have done before.

Participant 3, Age 42

Health Conditions

Two participants mentioned health conditions (migraines and high blood pressure) that precipitated a change from the COCP. Both reported problems with alternative methods of contraception and relied for some period of time on withdrawal methods, which, for one participant, resulted in an unintended pregnancy.

For peer review only - http://bmjopen.bmj.com/site/about/guidelines.xhtml

However, other participants had health conditions which had led to a change of contraception specifically for its health benefits. Endometriosis and menorrhagia were both drivers for considering the IUS. Another participant started a progestogen method to tackle Pre-menstrual syndrome.

Perimenopause

Confusion about the interplay between the menopause and hormonal contraceptive methods was common. Participants didn't understand how they would identify the menopausal transition while taking contraceptive methods that already affected their periods. This caused frustration as it could mean they were taking contraception even when it may no longer be needed to prevent pregnancy.

And I would love not to be on the pill. I'm assuming I'm gonna hit menopause soon. More than happy to. I'm embracing it. And it really is like, bring it on. Let me get on with the rest of my life. But how do you know, if you're taking the pill? How on earth do you know?

Participant 2, Age 50

Many participants spoke about perimenopausal symptoms and the difficulties they had experienced diagnosing and treating their symptoms. While some participants had been offered the IUS to control heavy periods, or as an alternative to the COCP, few were aware that the IUS could be used as a component of Hormone Replacement Therapy (HRT). Only one participant knew that combined hormonal contraception could alleviate perimenopausal symptoms. Overall there was a sense of frustration that their hormonal health had not been treated more holistically and that perimenopausal symptoms were misdiagnosed or ignored.

I don't think anyone has ever really spoken to me about how you might want to change contraception as you do approach menopause and why.

Participant 12, Age 48

Previous Experiences of Coercion and Trauma

Experiences of contraceptive coercion in a medical setting had severe and long-lasting effects. These incidents left participants feeling a loss of control over their own bodies and sense that their wishes had been violated. This undermined trust in both medical professionals and medical treatments. Traumatic experiences influenced the type of contraception that participants felt comfortable with, namely a method that was under complete user control such as condoms and pills. It also made participants hesitant about any methods that involve intimate procedures where they may experience a similar loss of autonomy and flashbacks. Complex and traumatic birthing experiences were also reasons for declining intrauterine contraception, or for requiring sedation in order to have one fitted.

Yeah, I hated that I felt really violated by almost, almost instantly. I felt like I'd been massively pressurized into it, and it was. I couldn't get it out again…I felt yeah really out of control…. And I've always been, since I had that injection, terrified of them. Anything hormonal.

Participant 13, Age 40 (discussing contraceptive injection as a university student)

Yeah, I mean even going for a smear is a big thing. I cried through most of them just because you're in that position. Somebody is going there. It's gonna hurt. I know they say it doesn't, but it, it does. I probably cry about it now, which is ridiculous, isn't it?

Participant 8, Age 52 (following failed induction of labour)

Conversely, a few participants reported their repeated exposure to medical procedures as enabling them to manage more intimate procedures, like the insertion of an intrauterine device, with ease. The stark difference

For peer review only - http://bmjopen.bmj.com/site/about/guidelines.xhtml

between these experiences seemed to centre around how much autonomy the participant felt they had during these procedures.

I had no problems, but I've done three rounds of IVF so I had so…sounds horrendous!...so many things put inside me, that actually this wasn't an issue.

Participant 14, Age 41

Other traumatic incidents outside the medical setting, including sexual assault and near miss attacks, were also reported and influenced choice of contraception. Again, this reinforced the need for control over their body and reproductive choices.

Yeah, I've had another really traumatic experience when I was 16 when somebody almost raped me and I think maybe I felt like it was really important to be totally in control

Participant 13, Age 40

DISCUSSION

Women over 40 have distinct motivators and barriers to choosing and using contraception. Previous studies have attributed the relatively low use of reliable contraception in women over 40 to women's perceptions of themselves as low risk due to declining fertility.(8,12) However, this study found that while women recognised that fertility levels decline in their 40s, they thought of pregnancy as a real possibility, and something that would have a large and detrimental impact on their lives. It created anxiety both for them as individuals, and also in their relationships.

Many participants reported stopping the COCP on the basis of age alone. This seemed to be provider led, but is not supported by clinical guidelines. In fact, combined hormonal contraception is increasingly recognised as a useful tool in women over 40 as it provides both contraception and stable levels of oestrogen, thereby treating perimenopausal symptoms.(17) Participants valued a tailored approach to contraceptive counselling and disliked being advised solely on the basis of age, which echoes the advice in clinical guidelines.(3,18–20) Despite this, women still feel they are not treated either holistically, or as individuals.

Many participants felt frustrated and disappointed that their healthcare provider had not helped them to identify perimenopausal symptoms earlier. Perimenopausal symptoms are not routinely discussed in contraception reviews meaning that these are missed, especially if women's periods are already altered by hormonal contraception.(21) Participants felt they would benefit from earlier information on the perimenopause and HRT in order to inform their contraceptive decisions.

Women over 40 are usually experienced in using contraception, but sometimes it is these experiences which create further obstacles to satisfactory contraception choice and use. side effects from previous contraception may make women more apprehensive to try new methods.(22–24) which is pertinent for women over 40 as they have had more exposure to adverse experiences over their reproductive lifetime. Interactions with healthcare providers in all areas of sexual and reproductive health coloured the contraception methods that women felt comfortable with. Experiencing coercion in a healthcare setting led to particularly strong negative feelings and narrowed contraceptive choices. These experiences undermined trust in methods that require provider insertion, which are typically the most long acting and reliable. Traumatic experiences outside a healthcare setting, such as sexual assault, also influenced women's method choice.

Online interviews allowed recruitment from areas of England and participants were able to participate across multiple healthcare localities and providers. However, online recruitment excluded women without access to the internet, and recruitment through social media platforms and narrowed the range of potential participants. This may have oversampled women with particularly difficult contraceptive needs or experiences, and those with higher anxiety about risk of pregnancy. Recruitment through specific social media platforms and interest groups may have also led to a relatively homogenous participant group that limited variety of themes discussed. Recruitment strategies to identify women with no digital access or who had additional needs such as translation,

For peer review only - http://bmjopen.bmj.com/site/about/guidelines.xhtml

may have provided additional insights. The interviews and analysis were conducted by a researcher with a clinical background, which may have been beneficial in interpreting their contraceptive history, but could have influenced the type of information women were comfortable sharing. Some participants may have been hesitant to share their negative experiences with healthcare providers or "non-medical" approaches to contraception, for example use of withdrawal methods or fertility tracking. The interviewer was a woman of comparable age to the participants which may have facilitated open discussion regarding personal experiences.

Clinicians and policymakers should recognise that contraception is still a priority for many women over 40. Contraceptive options should be assessed based on individual needs and risk factors, rather than by age alone. It is particularly important to engage in meaningful discussions about the risk and benefits of continuing with a combined hormonal method so that contraceptive options are not narrowed arbitrarily.(25) For women over 40, a reframing of contraception within a "hormonal health" consultation could allow more space for women to share their particular concerns and needs and would provide an opportunity to discuss the perimenopause and which methods could provide both contraception and relief of symptoms. Allotting appropriate time for contraceptive counselling would allow clinicians to ask about past experiences with contraceptives and previous traumatic experiences, both of which may influence the patient's contraceptive choice. These barriers are not traditionally addressed during contraceptive counselling.(26) While they are clearly important issues to address in every age group, it could be seen as particularly pertinent for women over 40 who are more likely to have been through traumatic births and invasive gynaecological investigations. Enquiring about these types of experiences when initiating or switching contraception would be a positive step in making the consultation more tailored and holistic.

Areas of future research could focus on how we meet the specific needs of women over 40 in practice, including how best to discuss risks and benefits of COCP with women over 40; how can we integrate perimenopause and contraceptive counselling effectively; and whether the principles of trauma-informed care can contribute to contraceptive counselling to widen the contraceptive choices for women over 40.

CONCLUSION

Women over 40 may be highly motivated to avoid pregnancy. This age group may have complex contraceptive histories with emerging perimenopausal symptoms. Women over 40 may have accumulated adverse experiences which impact their contraceptive choices. These factors need to be holistically explored by clinicians, to facilitate shared decision making.

ETHICS APPROVAL STATEMENT

Ethics approval was obtained from University College London (UCL) [Ethics no 19875/001].

COMPETING INTERESTS

None declared

FUNDING

Jo Burgin is a National Institute for Health Research academic fellow

CONTRIBUTORSHIP STATEMENT

JB and JoB devised the study. JoB designed the topic guide with input from JB. JoB carried out all interviews, transcription and analysis with supervision from JB. Emergent results were discussed with other researchers. JoB wrote the article with input from JB. Both authors approved the final version and are accountable for all aspects of the work.

DATA SHARING STATEMENT

For peer review only - http://bmjopen.bmj.com/site/about/guidelines.xhtml

Data are available upon reasonable request. Fully anonymised interview transcripts are available from Dr Jo Burgin, jo.burgin@nhs.net. Available for research purposes only.

For peer review only

For peer review only - http://bmjopen.bmj.com/site/about/guidelines.xhtml

REFERENCES

1. Ali M, Tran NT. Defining counselling in contraceptive information and services: outcomes from an expert think tank. BMJ Sex Reprod Health. 2022 Apr;48(2):79–81.

2. French RS, Geary R, Jones K, Glasier A, Mercer CH, Datta J, et al. Where do women and men in Britain obtain contraception? Findings from the third National Survey of Sexual Attitudes and Lifestyles (Natsal-3). BMJ Sex Reprod Health. 2018 Jan;44(1):16–26.

3. Roberts A, Noyes J. Contraception and women over 40 years of age: mixed-method systematic review. J Adv Nurs. 2009 Jun;65(6):1155–70.

4. Salisbury H. Helen Salisbury: The 10 minute appointment. BMJ. 2019 Jun 4;l2389.

5. Morrissey M, Shepherd E, Kinley E, McClatchey K, Pinnock H. Effectiveness and perceptions of using templates in long-term condition reviews: a systematic synthesis of quantitative and qualitative studies. Br J Gen Pract. 2021 Sep;71(710):e652–9.

6. FRSH. Faculty for Reproductive and Sexual Health, Clinical Guideline: Contraception for Women Aged over 40 Years (August 2017, amended September 2019). 2019.

7. ONS. Office for National Statistics, Conception Statistics, England and Wales, 2019. [Internet]. 2019. Available from: https://www .ons.gov.uk/peoplepopulationandcommunity/birthsdeathsandmarriages/conceptionandfertilityrates /datasets/conceptionstatisticsenglandandwalesreferencetables.

8. Sherman CA, Harvey SM, Noell J. "Are *They* Still Having Sex?" STIs and Unintended Pregnancy Among Mid-Life Women. J Women Aging. 2005 Sep 20;17(3):41–55.

9. Carslaw H, Cosh A. Contraception for older women. InnovAiT Educ Inspir Gen Pract. 2016 Aug;9(8):454–60.

10. Guerin J, Engelmann A, Mattamana M, Borgelt LM. Use of hormonal contraceptives in perimenopause: A systematic review. Pharmacother J Hum Pharmacol Drug Ther. 2022 Feb;42(2):154–64.

11. Daniels K, Mosher WD. Contraceptive methods women have ever used: United States, 1982-2010. Natl Health Stat Rep. 2013 Feb 14;(62):1–15.

12. Godfrey EM, Zapata LB, Cox CM, Curtis KM, Marchbanks PA. Unintended pregnancy risk and contraceptive use among women 45-50 years old: Massachusetts, 2006, 2008, and 2010. Am J Obstet Gynecol. 2016 Jun;214(6):712.e1-712.e8.

13. Harlow SD, Dusendang JR, Hood MM, Woods NF. Contraceptive preferences and unmet need for contraception in midlife women: where are the data? Womens Midlife Health. 2017 Dec;3(1):6.

14. Alspaugh A, Barroso J, Reibel M, Phillips S. Women's Contraceptive Perceptions, Beliefs, and Attitudes: An Integrative Review of Qualitative Research. J Midwifery Womens Health. 2020 Jan;65(1):64–84.

15. Braun V, Clarke V. Using thematic analysis in psychology. Qual Res Psychol. 2006 Jan;3(2):77–101.

16. Ritchie, J., & Lewis, J. Qualitative Research Practice—A Guide for Social Science Students and Researchers. London, Thousand Oaks, CA: Sage Publications Ltd.; 2003.

For peer review only - http://bmjopen.bmj.com/site/about/guidelines.xhtml

17. Mendoza N, Sanchez-Borrego R. Classical and newly recognised non-contraceptive benefits of combined hormonal contraceptive use in women over 40. Maturitas. 2014 May;78(1):45–50.

18. Kim CS, Tikhonov D, Merjanian L, Balica AC. Contraception pathway: application for midlife women. Womens Midlife Health. 2017 Dec;3(1):10.

19. Nelson AL, Cohen S, Galitsky A, Hathaway M, Kappus D, Kerolous M, et al. Women's perceptions and treatment patterns related to contraception: results of a survey of US women. Contraception. 2018 Mar;97(3):256–73.

20. Madden T, Secura GM, Nease RF, Politi MC, Peipert JF. The role of contraceptive attributes in women's contraceptive decision making. Am J Obstet Gynecol. 2015 Jul;213(1):46.e1-46.e6.

21. Marnocha SK, Bergstrom M, Dempsey LF. The lived experience of perimenopause and menopause. Contemp Nurse. 2011 Feb;37(2):229–40.

22. Godfrey EM, Chin NP, Fielding SL, Fiscella K, Dozier A. Contraceptive methods and use by women aged 35 and over: A qualitative study of perspectives. BMC Womens Health. 2011 Dec;11(1):5.

23. Gomez AM, Arteaga S, Aronson N, Goodkind M, Houston L, West E. No Perfect Method: Exploring How Past Contraceptive Methods Influence Current Attitudes Toward Intrauterine Devices. Arch Sex Behav. 2020 May;49(4):1367–78.

24. Alspaugh A, Reibel MD, Im EO, Barroso J. "Since I'm a little bit more mature": contraception and the arc of time for women in midlife. Womens Midlife Health. 2021 Dec;7(1):3.

25. Mendoza N, Soto E, Sánchez-Borrego R. Do women aged over 40 need different counseling on combined hormonal contraception? Maturitas. 2016 May;87:79–83.

26. Owens L, Terrell S, Low LK, Loder C, Rhizal D, Scheiman L, et al. Universal precautions: the case for consistently trauma-informed reproductive healthcare. Am J Obstet Gynecol. 2021 Aug;S0002937821008802.

For peer review only

For peer review only - http://bmjopen.bmj.com/site/about/guidelines.xhtml

Appendix 1

Discussion Guide: Factors affecting contraceptive choice in women over 40: A Qualitative Study

Confidentiality:
Are you somewhere we can speak in private?
Everything will be kept confidential unless you or someone else is in danger of harm.
We will use made up names in the report so you cannot be identified.
Please have your mobile on silent. Let me know if you would like a break or have to take a call. You may turn off video if you would feel more comfortable.

Explain consent form and gain consent through the chat function.

1. Participant background
Can you tell be a bit about yourself?
Why did you decide to help with this research project?
Age
Studies/work/training/qualifications
What area do you live in?
Do you live with anyone else?

2. Current contraceptive use
Do you need contraception at the moment?
Are you currently in a relationship?
What type of contraception do you usually use?
How have you decided what method of contraception to use?
What are your priorities when choosing a contraceptive method?
What are your main concerns when choosing a contraceptive method?
Do you feel your priorities and /or concerns have changed over time?

3. Information
Where do you get information about contraception?
Have you previously used online sources for contraception advice?
Do you feel you've been given enough information about your contraceptive method in the past?
Have you been given updated information about contraception?

4. Preventing pregnancy
Do you worry about unplanned pregnancy?
Is preventing pregnancy your primary concern when choosing a contraceptive method?
How would an unplanned pregnancy affect you at this point in your life?

5. Symptom control

For peer review only

Do you/have you experience/d any menopausal symptoms e.g. change in bleeding pattern, hot sweats, change in mood?

- If yes, how do they affect you?

Have you sought advice on how to manage these symptoms?

- If yes, where did you go and did you feel informed and supported?

Have you ever used a method of contraception to help manage these symptoms?

6. Your health

Have you ever had to change contraception because of your age or health?

Do you have any medical conditions that make choosing contraception more complicated?

For peer review only

"You're at that age now" Factors affecting contraceptive choice in women over 40

Standards for Reporting Qualitative Research (SRQR)*

http://www.equator-network.org/reporting-guidelines/srqr/

Title and abstract

	Page
**Title** - Concise description of the nature and topic of the study Identifying the study as qualitative or indicating the approach (e.g., ethnography, grounded theory) or data collection methods (e.g., interview, focus group) is recommended	1
**Abstract** - Summary of key elements of the study using the abstract format of the intended publication; typically includes background, purpose, methods, results, and conclusions	2

Introduction

**Problem formulation** - Description and significance of the problem/phenomenon studied; review of relevant theory and empirical work; problem statement	3
**Purpose or research question** - Purpose of the study and specific objectives or questions	3

Methods

**Qualitative approach and research paradigm** - Qualitative approach (e.g., ethnography, grounded theory, case study, phenomenology, narrative research) and guiding theory if appropriate; identifying the research paradigm (e.g., postpositivist, constructivist/ interpretivist) is also recommended; rationale**	3
**Researcher characteristics and reflexivity** - Researchers' characteristics that may influence the research, including personal attributes, qualifications/experience, relationship with participants, assumptions, and/or presuppositions; potential or actual interaction between researchers' characteristics and the research questions, approach, methods, results, and/or transferability	3
**Context** - Setting/site and salient contextual factors; rationale**	3
**Sampling strategy** - How and why research participants, documents, or events were selected; criteria for deciding when no further sampling was necessary (e.g., sampling saturation); rationale**	3
**Ethical issues pertaining to human subjects** - Documentation of approval by an appropriate ethics review board and participant consent, or explanation for lack thereof; other confidentiality and data security issues	11
**Data collection methods** - Types of data collected; details of data collection procedures including (as appropriate) start and stop dates of data collection and analysis, iterative process, triangulation of sources/methods, and modification of procedures in response to evolving study findings; rationale**	3

For peer review only

For peer review only - http://bmjopen.bmj.com/site/about/guidelines.xhtml

**Data collection instruments and technologies** - Description of instruments (e.g., interview guides, questionnaires) and devices (e.g., audio recorders) used for data collection; if/how the instrument(s) changed over the course of the study	3
**Units of study** - Number and relevant characteristics of participants, documents, or events included in the study; level of participation (could be reported in results)	3-4
**Data processing** - Methods for processing data prior to and during analysis, including transcription, data entry, data management and security, verification of data integrity, data coding, and anonymization/de-identification of excerpts	3
**Data analysis** - Process by which inferences, themes, etc., were identified and developed, including the researchers involved in data analysis; usually references a specific paradigm or approach; rationale**	3
**Techniques to enhance trustworthiness** - Techniques to enhance trustworthiness and credibility of data analysis (e.g., member checking, audit trail, triangulation); rationale**	3

Results/findings

**Synthesis and interpretation** - Main findings (e.g., interpretations, inferences, and themes); might include development of a theory or model, or integration with prior research or theory	4-8
**Links to empirical data** - Evidence (e.g., quotes, field notes, text excerpts, photographs) to substantiate analytic findings	4-8

Discussion

**Integration with prior work, implications, transferability, and contribution(s) to the field** - Short summary of main findings; explanation of how findings and conclusions connect to, support, elaborate on, or challenge conclusions of earlier scholarship; discussion of scope of application/generalizability; identification of unique contribution(s) to scholarship in a discipline or field	8-9
**Limitations** - Trustworthiness and limitations of findings	9

Other

**Conflicts of interest** - Potential sources of influence or perceived influence on study conduct and conclusions; how these were managed	9
**Funding** - Sources of funding and other support; role of funders in data collection, interpretation, and reporting	9

*The authors created the SRQR by searching the literature to identify guidelines, reporting standards, and critical appraisal criteria for qualitative research; reviewing the reference lists of retrieved sources; and contacting experts to gain feedback. The SRQR aims to improve the transparency of all aspects of qualitative research by providing clear standards for reporting qualitative research.

For peer review only - http://bmjopen.bmj.com/site/about/guidelines.xhtml

**The rationale should briefly discuss the justification for choosing that theory, approach, method, or technique rather than other options available, the assumptions and limitations implicit in those choices, and how those choices influence study conclusions and transferability. As appropriate, the rationale for several items might be discussed together.

Reference:
O'Brien BC, Harris IB, Beckman TJ, Reed DA, Cook DA. **Standards for reporting qualitative research: a synthesis of recommendations.** *Academic Medicine*, Vol. 89, No. 9 / Sept 2014 DOI: 10.1097/ACM.0000000000000388

For peer review only

For peer review only - http://bmjopen.bmj.com/site/about/guidelines.xhtml
